# Outcomes of ABO-incompatible kidney transplants with very high isoagglutinin titers: a single-center experience and literature review

**DOI:** 10.3389/fimmu.2024.1504495

**Published:** 2024-12-02

**Authors:** Hamza Naciri Bennani, Kadiatou Mamadou Bobo Barry, Johan Noble, Paolo Malvezzi, Thomas Jouve, Lionel Rostaing

**Affiliations:** ^1^ Nephrology, Hemodialysis, Apheresis and Kidney Transplantation Department, Grenoble University Hospital, Grenoble, France; ^2^ Grenoble-Alpes University, Grenoble, France

**Keywords:** kidney transplantation, isoagglutinins, ABO incompatible transplant, desensitization, apheresis, antibody-mediated rejection

## Abstract

**Background:**

ABO-incompatible kidney transplantation (ABOi-KTx) represents a possible solution to address the shortage of kidney donors. However, these transplants present immunological challenges, particularly when isoagglutinin titers are elevated pretransplant.

**Methods:**

Single-center retrospective study describing clinical and biological outcomes of 8 patients who underwent ABOi-KTx with initial isoagglutinin titers ≥ 1/512. All patients followed a desensitization protocol combining immunosuppression (rituximab, tacrolimus, mycophenolate mofetil, steroids), and specific or semi-specific apheresis sessions. Clinical and biological data were extracted from electronic medical records.

**Results:**

There were 5 males; median age of 62 years [34-82 years]; all achieved an isoagglutinin titer of ≤1/8 before transplantation after a median of 13 (range: 9-15) apheresis sessions. Three patients (37%) experienced acute humoral rejection, which required additional plasmapheresis sessions. Two patients developed chronic active rejection, successfully treated. On the infectious side, three patients developed BK-virus reactivation. Two patients developed cytomegalovirus viremia, and two others presented with bacterial infections. Surgically, two patients developed a lymphocele, and one had a perirenal hematoma. All patients survived the transplant with stable renal function: mean serum creatinine was 138 ± 15 µmol/L after four years of follow-up.

**Conclusion:**

ABO-incompatible kidney transplantation, even in patients with high isoagglutinin titers, is feasible and can achieve favorable long-term graft and patient survival outcomes. However, these procedures require substantial clinical expertise and close follow-up to monitor and manage the elevated risks of infection and rejection in this population.

## 1 Introduction

The number of living donor kidney transplants is increasing due to the shortage of kidneys from deceased donors and the growing number of patients on transplant waiting lists. In France, this shortage is exacerbated by increasing organ donation refusals, resulting in prolonged and variable wait times. ABO-incompatible (ABOi) kidney transplantation offers an opportunity to expand the donor pool and improve the survival prospects of patients awaiting a transplant (1, 2) especially in the absence of a national kidney paired donation program.

However, ABO incompatibility typically necessitates pre-transplant desensitization, involving apheresis and immunosuppression with rituximab, to reduce the risk of acute antibody-mediated rejection. Numerous studies have demonstrated that the long-term survival rates of patients and kidney allografts in ABOi transplants are comparable to those receiving ABO-compatible (ABOc) living donor transplants (1–15). Nevertheless, ABOi recipients are more prone to complications, such as hemorrhagic episodes related to apheresis, lymphocele, and BK virus infection (12, 16–22).

The presence of high isoagglutinin titers presents a significant challenge, increasing the risk of acute rejection and jeopardizing graft viability. This study aims to describe the clinical and biological outcomes of patients who underwent ABOi kidney transplantation with high isoagglutinin titers at a single center, with a particular focus on desensitization outcomes and related complications.

## 2 Patients and method

We conducted a retrospective, single-center observational study from January 2015 to July 2024; during that period there were 65 ABO incompatible kidney transplants of which eight had an initial isoagglutinin titer greater than 512. The objective of our study was to describe the clinical and biological outcomes of ABO-incompatible kidney transplant patients with high isoagglutinin titers following desensitization combining rituximab and apheresis.

### 2.1 Immunosuppression

Immunosuppression was initiated prior to transplantation. Rituximab (375 mg/m²) was administered 30 days before transplantation, and conventional immunosuppression began 15 days prior to transplantation, consisting of tacrolimus (0.05 mg/kg every 12 hours, targeting trough levels of 8-10 ng/mL), mycophenolic acid (MPA) (360 mg twice daily) or mycophenolate mofetil (MMF) (500 mg twice daily), and prednisone (0.5 mg/kg/day). In addition to these treatments, patients underwent apheresis sessions. Based on the initial isoagglutinin titers (IgM and IgG), and clinical profile, patients received one or more of the following:

- Semi-specific immunoadsorption (Globaffin^®^ column, Fresenius, Bad Homburg, Germany) with or without membrane filtration (Monet^®^, Fresenius Medical Care),
- Double filtration plasmapheresis (DFPP) performed on a PlasautoΣ with a Plasmaflo^®^ OP-08W and Cascadeflo^®^ EC-30W for the first session, followed by Cascadeflo^®^ EC-20W (Asahi Kasei Medical, Tokyo, Japan),
- Specific immunoadsorption (Glycorex^®^ column, Lund, Sweden, or ABO Adsopak^®^ column, Pocard, Russia),
- Plasma exchange using the Optia^®^ or Comtec^®^ monitor with fresh frozen plasma (FFP) the day before kidney transplantation.

**Table 1** compares the different apheresis techniques used.

**Table 1:** Apheresis techniques.

Technique	Description	Indications	Prescription	Advantages and Disadvantages
DFPP	Utilizes a two-step filtration system: - the first filter separates cellular elements from blood plasma. - the second filter removes plasma substances based on membrane pore size and molecular weight of the substance.	Purification role: IgG, IgM, Fibrinogen, alpha2-macroglobulin, LDL cholesterol, etc.	Treated PV = 1.5 x the patient’s PV Blood flow rate = 150 ml/min	Advantages: Semi-specific technique Low amount of substitution products Disadvantages: Hemorrhagic risk due to loss of coagulation factors Variable hemodynamic tolerance
Specific IA	Utilizes adsorption columns specifically targeting blood group antibodies (A or B).	Purification role: Isoagglutinins of IgG and IgM	Treated PV = 3 to 6 x the patient’s PV Blood flow rate = 50 ml/min	Advantages: Specific technique No hemorrhagic risk Good hemodynamic tolerance No substitution products Disadvantages: None noted
Semi-specific IA	Utilizes adsorption columns that remove IgG.	Purification role: IgG Addition of a Monet^®^ filter to remove IgM.	Treated PV = 100 ml/kg with a maximum of 10 liters Blood flow rate = 80 ml/min	Advantages: Semi-specific technique No hemorrhagic risk Good hemodynamic tolerance No substitution products Disadvantages: None noted
Plasma Exchange	Broadly removes plasma components, including isoagglutinins, by replacing the patient’s plasma with substitution product.	Purification and Transfusion roles: replacement with plasma providing coagulation factors.	Treated PV = 1.5 x the patient’s PV Blood flow rate = 80 ml/min if centrifugation and 150 ml/min if filtration Substitution = Plasma of the same blood group as the recipient (except if donor A and recipient B or vice versa) or AB plasma.	Advantages: Good hemodynamic tolerance Disadvantages: Non-specific Hemorrhagic risk unless plasma substitution prevents loss of coagulation factors Requires a substitution product

PV, Plasma Volume; DFPP, Double filtration plasmapheresis; IA, Immunoadsorption.

Apheresis sessions begin three weeks prior to the planned transplant date. Most patients initially undergo DFPP sessions, and, depending upon the decrease of isoagglutinin titers, specific immunoadsorption (IA) sessions may be used to achieve a more significant reduction in titers, with up to 15 liters of plasma treated in a single IA session (23). Semi-specific immunoadsorption is preferred for patients at risk of hypotension during sessions. The addition of a Monet^®^ filtration membrane, at least once a week depending on the IgM level, is essential for eliminating IgM isoagglutinin’s not removed by semi-specific IA (24). Each patient benefits from a personalized approach based on the kinetics of isoagglutinins measured before and after each apheresis session.

All patients underwent within 12 hours pretransplant a plasma exchange, which treated 1.5 times the plasma volume with 100% plasma replacement to mitigate the loss of coagulation factors during previous apheresis session (particularly DFPP ones) (25–28).

Extracorporeal circuit anticoagulation was performed using regional citrate anticoagulation during immunoadsorption, plasma exchange, or DFPP not coupled with hemodialysis. For DFPP coupled with hemodialysis, anticoagulation of the extracorporeal circuit was achieved with intravenous sodium heparin.

The goal was to achieve an isoagglutinin titer (IgG and IgM) of ≤ 1/8 on the day of transplantation.

In the posttransplant period we do not monitor isoagglutinin titers except when clinically necessary (i.e., drop in urine output or rise in serum creatinine level).

Induction therapy included basiliximab (20 mg on days 0 and 4). In cases where donor-specific antibodies (DSAs) were present, antithymocyte globulin (1 mg/kg daily for five days) was used instead of basiliximab. Post-transplant immunosuppression included tacrolimus (0.05 mg/kg every 12 hours, targeting trough levels of 8-10 ng/mL until day 30, then reducing to 5-8 ng/mL), MPA (720 mg twice daily) or MMF (1 g twice daily), administered until day 15, after which doses were halved, and steroids (methylprednisolone 10 mg/kg on day 0, with a maximum of 500 mg, 6 mg/kg on day 1, 4 mg/kg on day 2, 2 mg/kg on day 3, 1 mg/kg on day 4, followed by prednisone at 0.5 mg/kg on day 5, 0.25 mg/kg on day 6, then 10 mg daily until day 90, and finally 5 mg daily).

A systematic kidney biopsy is performed at three- and twelve-months post-transplant. Otherwise, the indications for kidney biopsy remain the same as for ABO-compatible transplants.

### 2.2 Prophylaxes

If the donor was CMV-seropositive and the recipient was CMV-seronegative, or if the recipient was CMV-seropositive, valganciclovir (900 mg daily, adjusted for estimated glomerular filtration rate [eGFR]) was administered for six or 3 months, respectively. For Pneumocystis jirovecii prophylaxis, sulfamethoxazole/trimethoprim (400 mg/80 mg every day) was given for six months.

### 2.3 Collected data and statistical analyses

Clinical and biological data were collected from electronic medical records using the CristalNet and Easily software systems. Statistical analyses were performed using Excel 2016 and R statistical software. Quantitative variables are presented as means ± standard deviations (SD) or medians with quartiles (Q1–Q3), while qualitative variables are presented as numbers and percentages.

The study was conducted in accordance with the guidelines of the Declaration of Helsinki and was approved by the French National Committee for Data Protection (CNIL; approval number 1987785v0). The biobank collection number is BRIF BB-0033-00069. Informed consent was obtained from all participants in the study.

## 3 Results

We included eight patients, i.e., 12.3% of our ABO incompatible cohort with a male-to-female ratio of 5:3. The median age at transplantation was 62 years (range: 34-82 years). The median posttransplant follow-up duration was 60 (range: 4-96) months. All patients were undergoing their first kidney transplant. Rituximab was administered at a dose of 375 mg/m² on day -30 to all patients, except two who received an additional rituximab infusion on day -15 (375 mg/m²) due to the presence of donor-specific antibodies prior to transplantation. The characteristics of the patients are presented in **Tables 2**, **3**.

**Table 2:** Characteristics of Patients.

	Patients (n=8)
Donor Age (years)	60 ± 13
Donor measured GFR (mL/min)	79 ± 15
Etiology of ESKD	
Vascular nephropathy (%)	4 (50%)
ADPKD (n)	3 (37%)
Diabetes nephropathy (n)	1 (13%)
ABO incompatibility	
A → O	7 (87%)
AB → O	1 (13%)
Isoagglutinin titers (medians)	
Before Rituximab	
Anti-A IgM	64 [32;512]
Anti-A IgG	1024 [256;2048]
Anti-B IgM	128
Anti-B IgG	1024
Before apheresis	
Anti-A IgM	64 [16;128]
Anti-A IgG	512 [32;1024]
Anti-B IgM	128
Anti-B IgG	256
After kidney transplantation	
Anti-A IgM	
M+1	8 [2;32]
M+3	8 [2;64]
M+6	4 [2;8]
M+12	4 [1;8]
Anti-A IgG	
M+1	32 [4;512]
M+3	32 [4;2048]
M+6	16 [4;128]
M+12	32 [2;64]
Anti-B IgM	
M+1	2
M+6	2
Anti-B IgG	
M+1	4
M+6	4
HLA mismatches	
Class I (A/B/C)	3.7 ± 1.8
Class II (DR/DQ/DP)	4 ± 2
Anti-HLA antibodies (n ;%)	4 (50%)
Blood transfusion	2 (25%)
Pregnancy	2 (25%)
DSA (%)	2 (25%)
MFI	DQ2 at 800 and A32 at 4600
Cold ischemia time (min)	78 ± 20
Induction Therapy (n; %)	
ATG	2 (25%)
Basiliximab	6 (75%)
Preemptive kidney transplantation	1 (13%)

ESKD, end-stage kidney disease; CGN, chronic glomerulonephritis, HLA, human leukocyte antigen; GFR, glomerular-filtration rate; ATG, antithymocyte globulins; ADPKD, Autosomal dominant polycystic kidney disease; MFI, mean fluorescent intensity; DSA, Donor-specific antibodies.

**Table 3:** Individualized characteristics of patient.

Patient	Donor/Recipient ABO incompatibility	Isoagglutinin titers (anti-A IgG; anti-A IgM; anti-B IgG; anti-B IgM)							Number of apheresis sessions	Acute rejection	Serum creatinine and eGFR at the last follow-up (µmol/L; ml/min/1,73m²)	Follow-up duration in months
		Before rituximab	Before apheresis	The day of kidney transplan tation	After Kidney transplantation							
					M1	M3	M6	M12				
1	A/O	2048; 512	2048; 128	4; 1	512; 32	2048; 64	NA	NA	15 (11 IAss, 3 IAs,1 PE)	No	130; 59	3
2	A/O	2048; 256	512; 64	4; 1	64; 4	128; 8	128; 8	64; 8	15 (13 DFPP, 1 IAs, 1 PE)	No	117; 70	12
3	AB/O	256; 32; 1024; 128	32; 16; 256; 128	2; 1; 4; 2	32; 8; 4; 2	NA	32; 8; 4; 2	NA	14 (8 IAss, 5 IAss + Monet^®^, 1 PE)	No	100; 53	30
4	A/O	1024; 32	512; 16	8; 1	32; 16	32; 2	16; 4	32; 4	9 (6 DFPP, 2 IAs, 1 PE)	Yes	134; 35	48
5	A/O	1024; 256	256; 128	8; 2	16; 8	64; 8	32; 8	16; 2	12 (11 DFPP, 1 PE)	Yes	147; 41	60
6	A/O	1024; 64	1024; 128	8; 2	4; 2	8; 4	4; 4	4; 2	10 (9 DFPP, 1 PE)	No	122; 42	72
7	A/O	512; 64	256; 32	4; 1	4; 2	8; 4	4; 2	2; 1	10 (7 DFPP, 2 IAs, 1 PE)	No	150; 30	72
8	A/O	1024; 64	1024; 64	2; 1	16; 4	8; 4	16; 4	32; 8	14 (10 DFPP, 3 IAs, 1 PE)	Yes	113; 71	12

GFR, glomerular-filtration rate; IAss, semi-specific immunoadsorption; IAs, specific immunoadsorption; DFPP, Double filtration plasmapheresis; PE, plasma exchange; NA, Not available.

**Figure 1:**
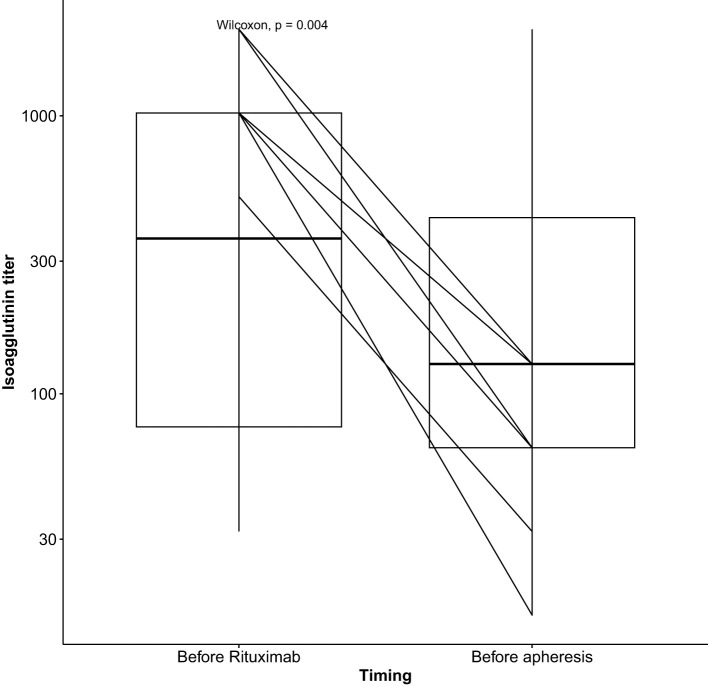
Outcomes of isoagglutinin titers after rituximab infusion.

**Figure 2:**
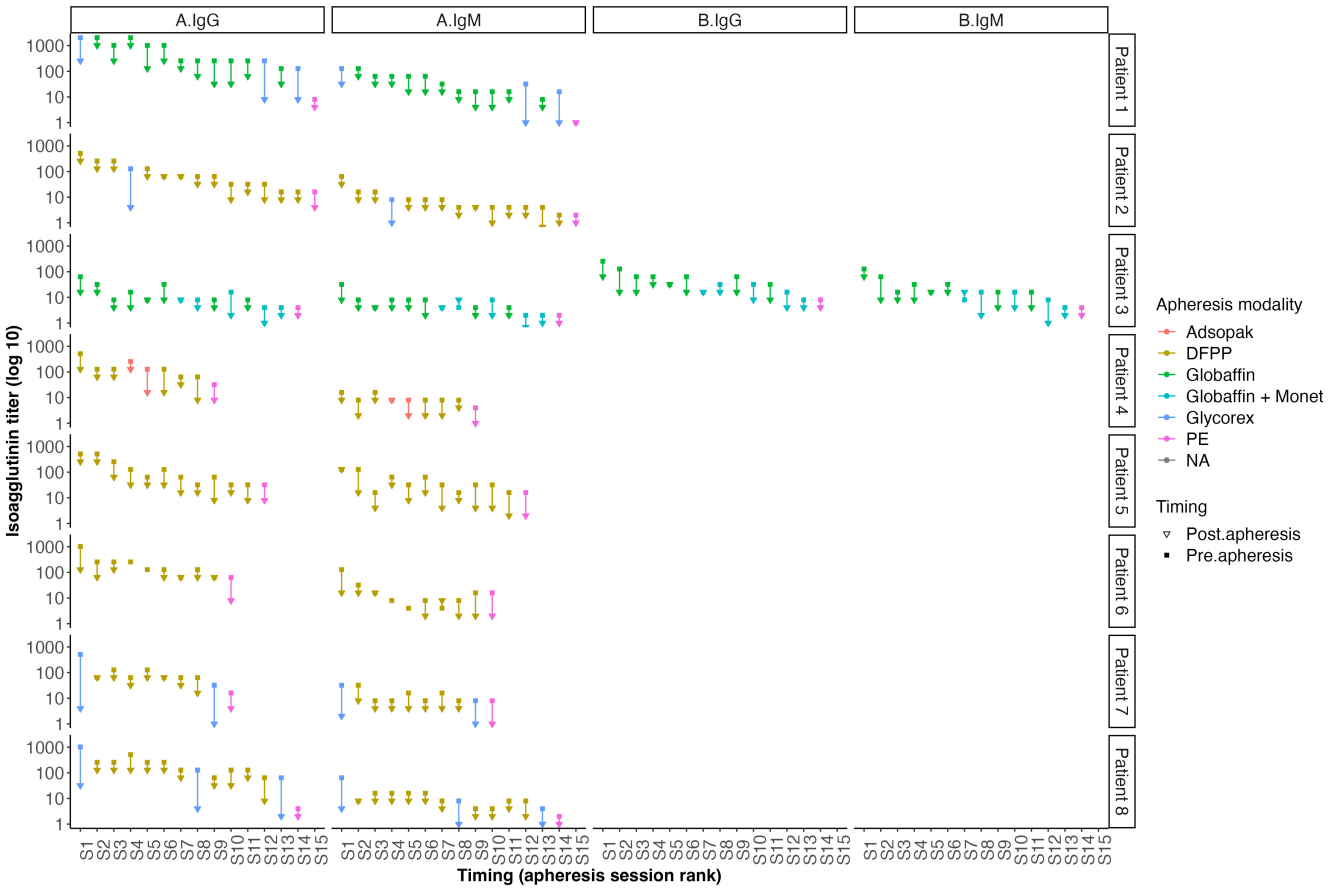
Outcomes of isoagglutinin titers after each apheresis session according to the technique used.

Isoagglutinin levels decreased significantly following the rituximab infusion and prior to the initiation of apheresis, as shown in **Table 2** and **Figure 1**. The target isoagglutinin level of less than 1/8 was achieved in all patients before kidney transplantation after a median of 13 (range: 9-15) apheresis sessions (**Figure 2**).

**Figure 3:**
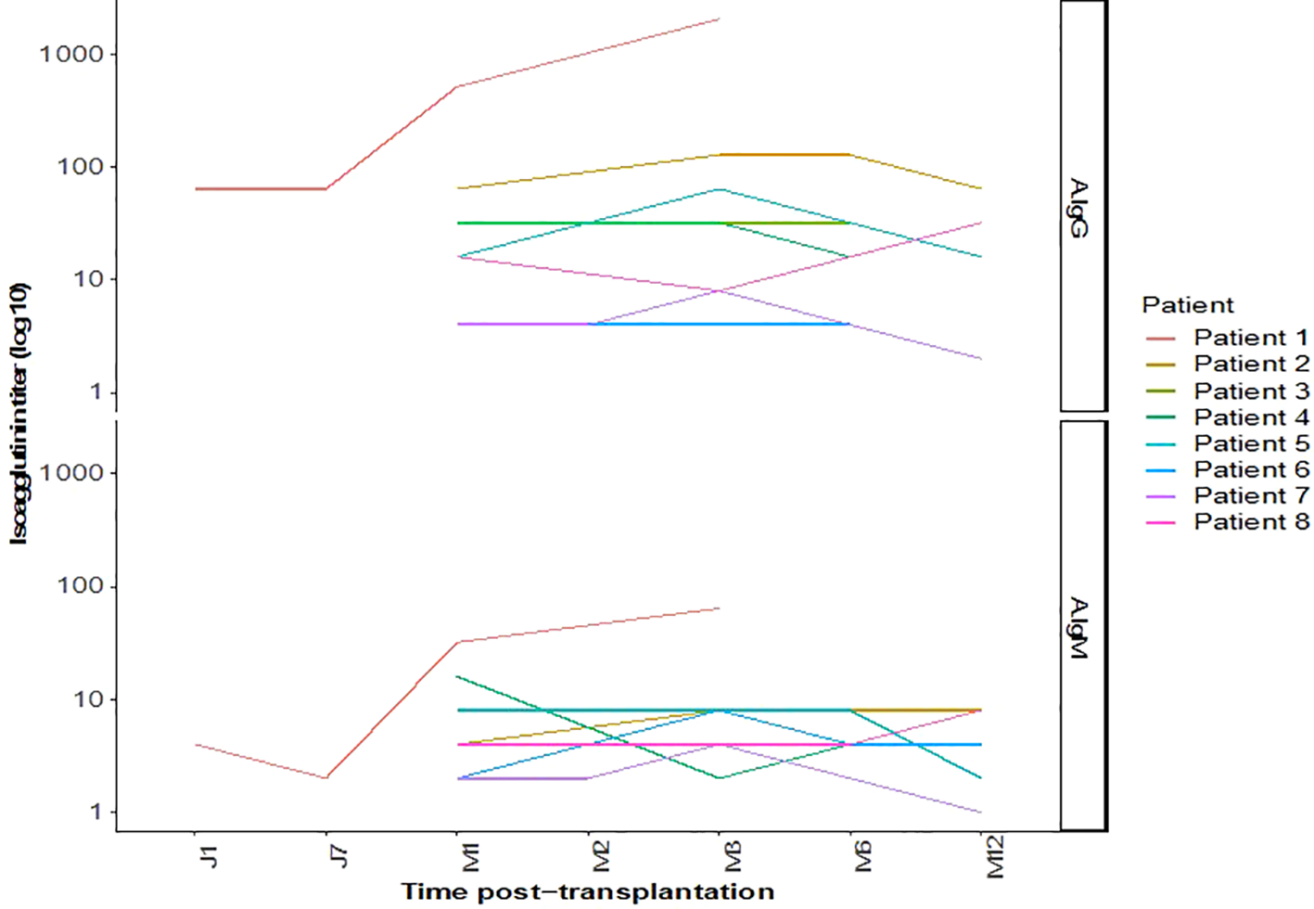
Outcomes of isoagglutinin titers after kidney transplantation.

**Figure 4:**
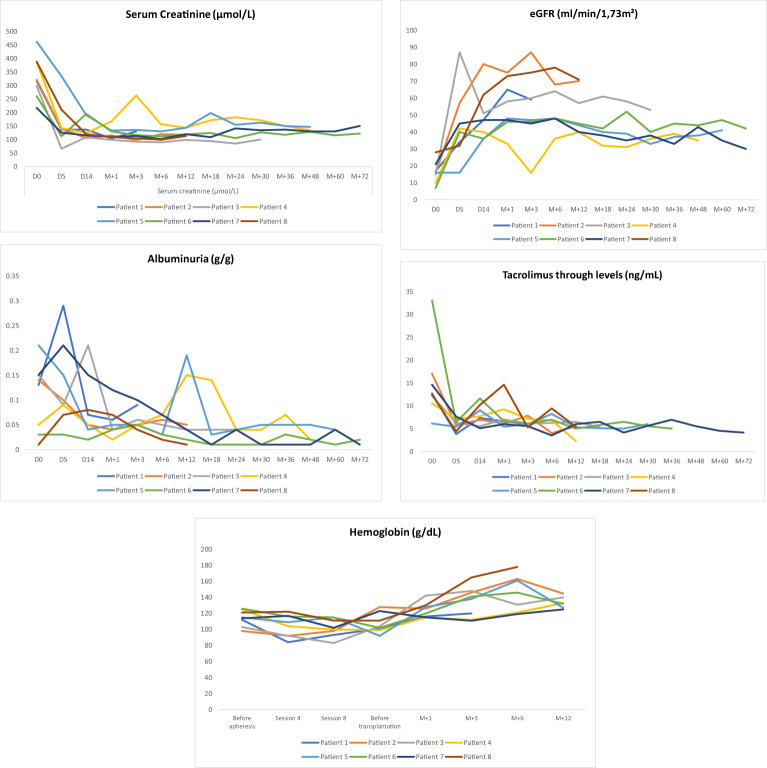
Outcomes of Albuminuria (g/g), eGFR(ml/min/1,73m^2^), tacrolimus through levels (ng/mL), Serum creatinine (μmol/L) and Homoglobin (g/dL) during follow-up.

All the patients experienced immediate graft function; however, in 3 of them as of postoperative day (POD) 2 there was a decrease in urine output while serum creatinine was plateauing at > 250 µmol/L. This was highly suggestive of acute humoral rejection because the allograft doppler ultrasound analyses were normal. For these three patients, the anti-A IgG isoagglutinin level had risen to 1/32 in two patients and 1/16 in the third (i.e., rebounds), while anti-A IgM levels remained below 1/8. This required resumption of plasma exchange (4, 5, and 8 sessions, respectively), leading to an immediate increase of diuresis and improvement in renal function (Patient 4, 5 and 8 in **Table 3** and **Figures 3**, **4**). One of these patients also presented with elevated creatinine (280 µmol/L) at the 1-month follow-up, which prompted a graft biopsy. The biopsy revealed mixed humoral and cellular rejection (grade 3), which was successfully treated with methylprednisolone boluses, four plasma exchange sessions, and a single dose intravenous immunoglobulin (IVIg) -20 gr- after the last apheresis session.

During follow-up, two other patients (2 women, each having her husband as a donor) developed chronic active humoral rejection at 6 and 12 months, respectively, confirmed by graft biopsy after observing a rise in plasma creatinine. Both of them had very low isoagglutinin titers at posttransplant and did not experience any episodes of acute rejection. One patient was ABOi plus HLA incompatible (DSA at pretransplant: anti-A32 with MFI of 1,200): the 6-month protocol biopsy showed evidence for chronic active antibody-mediated rejection: she was therefore placed on tocilizumab therapy (162 mg/week subcutaneously for one year). The second patient developed a *de novo* DSA by 12 months posttransplant (anti-DQ7 with MFI at 2,000). She was treated by a single dose of Rituximab (1gr).

The outcomes for serum creatinine, estimated glomerular filtration rate (eGFR), albuminuria, and tacrolimus trough levels are illustrated in **Figure 4**.

**Figure 5:**
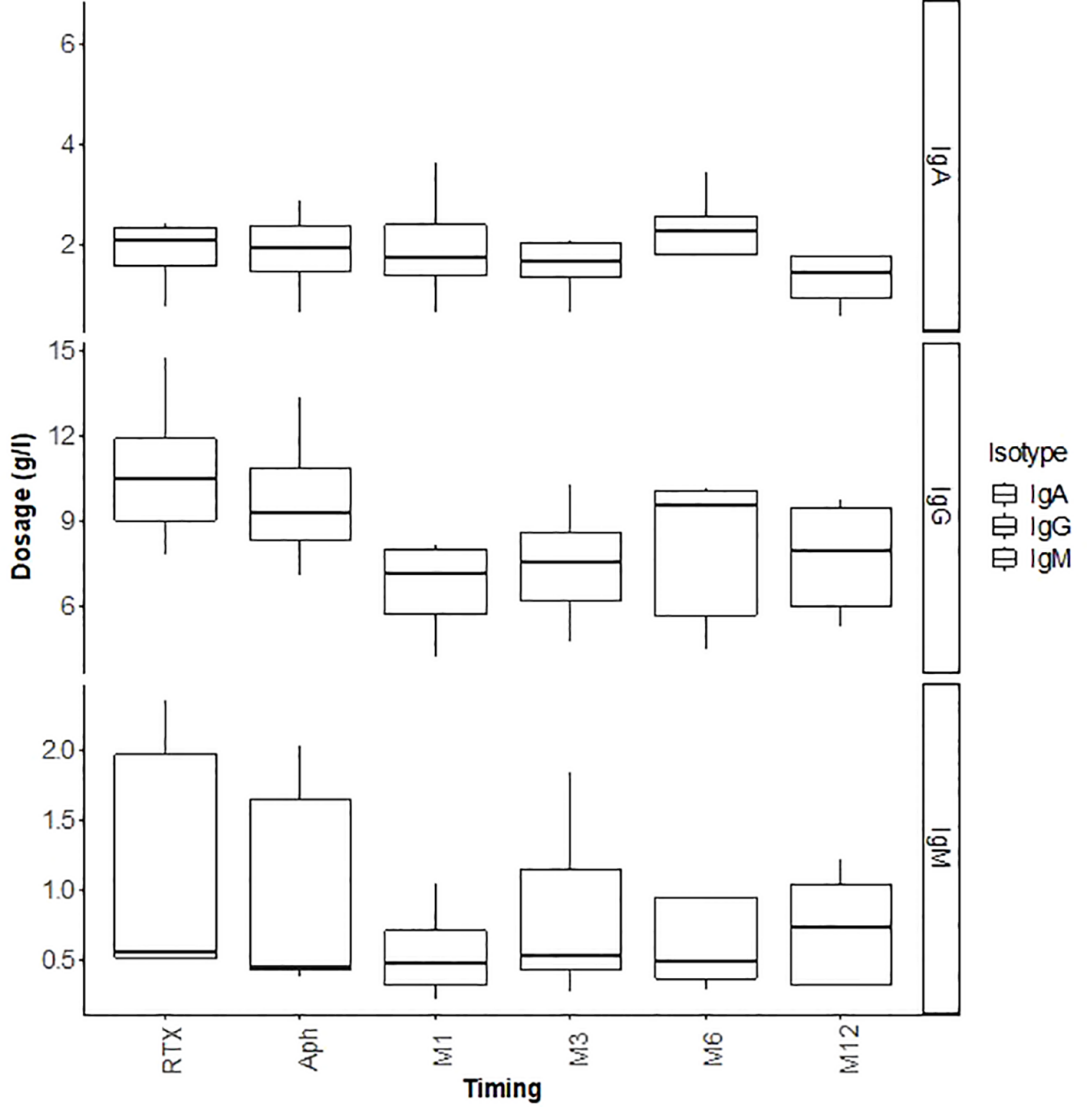
Outcomes of immunoglobulins (IgG, IgM, IgA) in g/L during the first 12 months post-transplant.

Regarding infectious complications, three patients developed BK virus viruria at months 1, 5, and 6, with two showing positive BK viremia and one presenting with BK virus nephropathy on a graft biopsy at month 3 (**Table 4**). Management involved reducing immunosuppression by lowering tacrolimus target trough levels, substituting mycophenolate mofetil with everolimus, and administering every two weeks IVIg (20 gr) for three months. The outcome was favorable, with resolution of BK viremia and viruria, and disappearance of BK virus nephropathy on follow-up biopsy at month 12. Among these three patients, one also developed concurrent CMV viremia and acute pyelonephritis; both conditions were successfully managed with valganciclovir and antibiotics with favorable outcomes. An additional patient developed CMV viremia, which also responded well to a three-week course of valganciclovir. Lastly, one patient developed a bacterial infection (community-acquired pneumonia) which resolved with antibiotic therapy. None of the patients developed hypogammaglobulinemia secondary to rituximab after one year of follow-up (**Figure 5**).

**Table 4:** Outcomes and complications of patient and allograft post-transplantation.

	Patients (n=8)
Patient Survival at last follow-up	8 (100%)
Graft survival at last follow-up	8 (100%)
Delayed graft function (serum creatinine > 250 µmol/L at D5)	2 (25%)
Hemodialysis at posttransplant	0
Serum creatinine (µmol/L) D0 D5 D14 M+1 M+3 M+6 M+12 M+18 M+24 M+36 M+48	331 ± 78 158 ± 81 136 ± 35 120 ± 22 131 ± 55 115 ± 22 121 ± 16 139 ± 43 133 ± 38 138 ± 28 138 ± 15
Acute humoral rejection	3 (37,5%)
Acute cellular rejection	1 (12,5%)
Chronic humoral rejection	2 (25%)
Lymphocele (n; %)	2 (25%)
Hematoma requiring surgical revision (n; %)	1 (12,5%)
Patients requiring red-blood cell transfusion between D0 and D5 (n; %)	2 (25%)
BKV viruria (n; %) BKV viremia (n; %) BKV nephropathy (n; %)	3 (37,5%) 2 (25%) 1 (12,5%)
CMV viremia (n; %)	2 (25%)
Acute pyelonephritis (n; %)	1 (12,5%)
Bacterial pneumopathy (n; %)	1 (12,5%)

BKV, BK virus; CMV, cytomegalovirus.

Surgically, two patients developed lymphocele, which resolved spontaneously. One patient presented with a compressive perirenal graft hematoma and required surgical revision on day 14 post-transplantation. Additionally, two patients required transfusion with two units of red blood cells (RBCs) immediately post-transplant due to low hemoglobin level without external bleeding (**Table 4**). Hemoglobin levels during the first year posttransplant are depicted in **Figure 4**.

## 4 Discussion

In this study we demonstrate that it is feasible to perform ABO incompatible kidney transplantation even when isoagglutinin titers are very high after a median of 13 (range: 9-15) apheresis sessions pretransplant; it resulted with 100% patient and graft survival in the long term. However, in such situations the risk of antibody (isoagglutinin)-mediated rejection and infectious complications remains high and therefore such hazardeous transplant should only be performed in well-experienced centers.

Chung et al. (29), in a comparative study of ABOi KTx patients with either high titers (≥1:256, n=8) or low titers (≤1:128, n=6), found that the high-titer group required more i) pre-transplant apheresis sessions (10.5 ± 3.5 vs. 6.0 ± 1.3; p = 0.01) to achieve an acceptable titer before transplantation, and ii) post-transplant sessions (1.6 ± 1.8 vs. 0 ± 0) due to a rebound in isoagglutinin levels compared to the low-titer group. Indeed, the rebound of isoagglutinin titers within the first two weeks after kidney transplantation can be a risk factor for rejection, as demonstrated by Süsal et al. (30) in a case of ABOi kidney transplantation (A donor, O recipient) with initial isoagglutinin titers of 1/124 for IgM and 1/1024 for IgG, which were reduced to <1/8 after desensitization. However, she presented a typical humoral rejection with a rebound of IgG titers to 1/36 and of IgM to 1/8 on POD5; she did not respond to methylprednisolone pulses and plasmapheresis. Nonetheless, the acute rejection was controlled by IV daratumumab combined with four specific immunoadsorption sessions.

Won et al. (31) showed that predictive factors for the rebound of isoagglutinin titers after kidney transplantation included a short interval (<7 days) between rituximab administration and the first plasmapheresis, a high initial titer (≥256), low rate of titer reduction, and blood group O. They also demonstrated that low-dose rituximab (200 mg) had no significant effect on isoagglutinin rebound titers but allowed for a reduction in infection rates. Rarely, despite intensifying apheresis sessions, kidney transplantation may fail due to incomplete reduction of isoagglutinin titers, as shown by Wilpert et al. (32), who were unable to transplant 4 out of 11 patients with initial titers ≥ 1/256.

ABO-incompatible kidney transplantation represents a major advancement in transplant medicine, and should no longer be viewed as a barrier to expanding the organ donor pool (1, 2, 13). Theoretically, the number of kidney transplants from living donors can be increased by up to **30%** when patients are transplanted across the ABO antibody barrier (33). Nevertheless, it poses significant immunological and infectious challenges, particularly in patients with elevated isoagglutinin titers, as demonstrated by our study. This underscores the importance of rigorous long-term monitoring and individualized therapeutic adjustments to ensure optimal outcomes.

### 4.1 Desensitization protocol and management of isoagglutinin titers

Pre-transplant desensitization is essential for the success of ABO-incompatible transplantation. In our cohort, Apheresis sessions begin three weeks prior to the planned transplant date. Isoagglutinins measured before and after each apheresis session. All patients achieved an isoagglutinin titer below 1/8 prior to transplantation, demonstrating the effectiveness of the desensitization protocol combining rituximab and apheresis (3). However, 37% of them developed acute antibody-mediated rejection (AMR), which coincided with a rise in isoagglutinin titers, necessitating additional plasmapheresis. These interventions resulted in a favorable outcome.

In the posttransplant period we typically do not monitor isoagglutinin titers unless clinically necessary (i.e., drop in urine output or rise in serum creatinine level). If there are no complications, patients typically remain hospitalized for seven days post-transplant.

### 4.2 Acute and chronic rejections: immunological challenges

Humoral and cellular rejections pose significant threats to graft survival in ABO-incompatible (ABOi) transplant patients. Despite achieving acceptable isoagglutinin titers pre-transplant, 37% of patients in our cohort developed acute antibody-mediated rejection (AMR) post-transplant, while 25% experienced active chronic AMR during follow-up. These cases required additional treatments, such as tocilizumab (a monoclonal antibody that blocks IL-6 receptor) in one patient and a second rituximab infusion in another, which stabilized graft function.

These findings are consistent with those of Gan et al. (3), who reported a high incidence of acute cellular rejection (12.5%) and acute humoral rejection (8.3%) in a cohort of 26 ABOi kidney transplant patients with pre-desensitization IgG titers ranging from 2 to 2048. Similarly, Hew et al. (4) reported that 18.2% of pediatric ABOi kidney transplant recipients experienced acute cellular rejection within the first 12 months post-transplant. In addition, Chung et al. (29), in a comparative study of ABOi patients with high titers (≥1:256, n=8) and those with low titers (≤1:128, n=6), found a higher incidence of acute cellular rejection in the high-titer group (37% vs. 0%).

The overall incidence of acute rejections appears to be higher in ABOi transplants compared to ABO-compatible (ABOc) transplants. De Weerd et al. (13), in a study comparing 296 ABOi kidney transplant recipients with 1184 ABOc living donor and 1184 ABOc deceased donor kidney transplant (KTx) recipients, found acute rejection rates of 29%, 18%, and 19%, respectively (p = 0.001). However, this did not significantly impact graft or patient survival. In support of this, Deng et al. (8) demonstrated that the presence of pre-transplant donor-specific antibodies (DSA) significantly increased the risk of acute antibody-mediated rejection and graft loss in both ABOi and ABOc kidney transplants (8).

The use of B-cell depleting agents like rituximab plays a crucial role in reducing the risk of humoral rejection, as shown in a study by Bleasel et al. (34). They compared 66 ABOi KTx patients who did not receive B-cell depletion to 18 ABOi KTx patients treated with rituximab. They observed humoral rejection in 39% of patients without rituximab versus in only 6% of patients who received rituximab within the first 3 months posttransplant. Additionally, 6 patients without rituximab required splenectomy for refractory rejection, with two cases of early graft loss due to humoral rejection on POD 6 and extensive graft necrosis. By comparison, in our series of 44 ABOi KTx recipients in which all have had received before transplantation rituximab infusion we did not observe a single graft loss (6, 7).

Long-term management of ABOi kidney transplants remains challenging due to the risk of chronic rejection. Chronic rejection remains a leading cause of graft loss, particularly in HLA-incompatible transplants, even in the presence of residual antibody titers (4). In our cohort, we observed two cases of chronic rejection.

Guy et al. (5) reported that long-term histological lesions, i.e., after 5 years of follow-up were similar between ABOi and ABOc KTx patients. They also found that microvascular inflammation was less severe in ABOi KTx patients without DSA compared to both ABOi and ABOc KTx patients with DSA, supporting the theory that accommodation may mitigate the harmful effects of residual isoagglutinins and prevent chronic lesions. Tasaki et al. (35) demonstrated that ABOi patients exhibited downregulation of donor-specific blood group antibodies while continuing to produce antibodies against other antigens. Finally, Heo et al. (15), in a study of 1292 ABOc and 347 ABOi kidney transplants, showed that ABOi transplants are associated with a lower risk of *de novo* DSA production and chronic AMR.

These data confirm that ABOi kidney transplants can be safely performed, although they require both short-term (particularly during the first six months, when most acute rejections occur) and long-term follow-up strategies. Personalized immunosuppressive adjustments are critical to preventing post-transplant immunological complications.

### 4.3 Infectious complications: impact of immunosuppression

Infectious complications represent a major challenge in the management of ABO-incompatible (ABOi) transplant patients. In our cohort, three patients developed BK virus (BKV) viruria, two of whom had positive BKV viremia, and one case of BKV-associated nephropathy (BKVAN) was confirmed by biopsy. The management of these infections required a reduction in immunosuppression, including lowering the target tacrolimus levels to *between 3 and 4 ng/mL*, substituting mycophenolate mofetil with everolimus (*target trough level of 6 to 7 ng/mL*), and administering intravenous immunoglobulins (IVIg), i.e., it has been shown that IVIg do contain specific anti-BKV antibodies (36). These approaches are consistent with recommendations in the literature (37). In our three patients the outcome was favorable, with BKV viremia and viruria resolving and BKVAN disappearing in the graft biopsy performed 12 months after initial diagnosis.

The incidence of viral infections, particularly BKV, is well-documented in ABOi kidney transplants. Sharif et al. (21) found that ABOi KTx patients had a significantly higher incidence of BKVAN compared to HLA-incompatible (HLAi) KTx patients (17.7% vs. 5.9%, p = 0.008). Eder et al. (12), in a study of 465 patients (42 ABOi, 106 HLAi, and 317 ABOc/HLAc controls), showed that ABOi patients had significantly higher Torque Teno Virus (TTV) loads than HLAi KTx patients and controls at 3- and 6-months post-transplant, reflecting the degree of immunosuppression. As a result, biopsy-proven BKVAN was more frequent in ABOi patients compared to HLAi and control patients (11.9% vs. 2.8% vs. 4.1%; p = 0.046). Moreover, ABOi patients treated with rituximab had higher TTV viral loads at 3 months compared to those who did not receive rituximab. This suggests that rituximab significantly increases the risk of BKV infection, as confirmed by a meta-analysis of 4256 ABOi patients conducted by Lee et al. (38). The study found that higher doses of rituximab (>500 mg) were associated with a higher risk of BKV infections compared to lower doses (200 mg), with no significant differences in rejection rates or graft function.

Intense immunosuppression, rather than an intrinsic characteristic of ABOi transplants, likely contributes to the increased risk of infections. In a 2018 study (39), rituximab was responsible for severe hypogammaglobulinemia (IgG < 4 g/L) in 25% of ABOi patients within the first-year post-transplant, necessitating IVIg infusions. However, the use of IVIg led to an infection rate comparable to that of ABOi patients with mild to moderate hypogammaglobulinemia who did not receive IVIg, highlighting the importance of regular IgG monitoring.

Pre-transplant isoagglutinin titers also seem to influence infection risk. In a study of 48 ABOi KTx recipients (19% with titers ≥ 1/256) compared to 96 ABO-compatible (ABOc) KTx recipients, Speer et al. (40) found that ABOi patients with high titers (≥1:256) had a higher incidence of BKV replication than those with low titers or ABOc patients. Koo et al. (1) similarly observed that ABOi patients with low titers (≤1:64) had fewer bacterial infections than those with high titers (≥1:128; p = 0.022), likely because patients with high titers require more aggressive desensitization and immunosuppression.

Interestingly, bacterial infections were less common, likely due to systematic antibiotic prophylaxis with sulfamethoxazole/trimethoprim and/or phenoxymethylpenicillin during the first 6 months post-transplant. We observed, in our series, only one case of bacterial pneumonia and one case of urinary tract infection (UTI) during the follow-up period. However, Speer et al. (40) reported that ABOi KTx recipients developed UTIs (22.9% vs. 8.5%; p = 0.019) and pneumonia (8.3% vs. 1.0%; p = 0.025) more frequently than ABOc KTx patients.

To mitigate infection risk, selective apheresis may offer some protection, as shown by Matuschik et al. (41). Their study comparing ABOi patients desensitized using specific immunoadsorption (IA) with Glycosorb^®^ versus non-specific IA with Immunosorba^®^ found that non-specific IA significantly increased the risk of severe postoperative infections, mainly of urinary origin (adjusted HR 3.08, 95% CI: 1.3–8.1).

These findings emphasize the need to strike a delicate balance between immunosuppression to prevent rejection and minimizing infection risk. Continuous optimization of prophylaxis protocols and individualized immunosuppression management are crucial to achieving this balance.

### 4.4 Surgical complications and hemorrhage management

Although less common, surgical complications can still pose challenges in the management of ABO-incompatible (ABOi) kidney transplants. In our study, two patients (25%) developed a lymphocele. The significantly higher incidence of lymphoceles among ABOi KTx patients, compared to ABO-compatible (ABOc) recipients, has been supported by Habicht et al. (20) and corroborated by a study we conducted in 2016, which included 44 ABOi and 44 ABOc KTx patients (6, 7), where 19% of ABOi patients developed a lymphocele.

One potential explanation for the increased incidence of lymphocele is the impact of mycophenolate mofetil (MMF), as demonstrated by Lopau et al. (42). It is hypothesized that the use of MMF one to two weeks prior to kidney transplantation surgery in ABO-incompatible patients may heighten the risk of lymphocele formation. Another hypothesis pertains to the necessity of preoperative apheresis sessions. Jänigen et al. (43) found that undergoing eight or more sessions of immunoadsorption/plasmapheresis preoperatively significantly increases the risk of developing a lymphocele.

From a hemorrhagic standpoint, one patient (12.5%) in our study required surgical revision due to a perirenal hematoma. The literature shows a higher risk of bleeding complications in ABO-incompatible (ABOi) kidney transplant (KTx) recipients compared to ABO-compatible (ABOc) recipients. For instance, a meta-analysis by de Weerd et al. (44) found a significantly higher incidence of postoperative hemorrhagic complications in ABOi patients (11%) vs. ABOc patients (4%) (p < 0.001). Similarly, a study by Zschiedrich et al. (45) comparing 97 ABOi KTx to 107 ABOc KTx identified bleeding complications in 21% of ABOi patients compared to 13% of ABOc patients (p = 0.19). Lastly, Habicht et al. (20) reported bleeding events in 9.5% of ABOi recipients vs. 2% in ABOc recipients.

These hemorrhagic events are often linked to the depletion of coagulation factors during apheresis sessions (17–19, 29), a process required for ABOi desensitization, particularly for patients with high antibody titers (≥1:256), which necessitates more intensive apheresis. To counteract coagulation factor loss (25–28), our protocol involved a pre-transplant plasma exchange, where 1.5 times the plasma volume was treated with 100% plasma replacement to replenish coagulation factors. Although our study sample size limits robust conclusions, the data suggest that targeted plasma exchange could play a role in managing bleeding risks in ABOi patients. However, further research is necessary to confirm its efficacy and safety across larger patient cohorts.

### 4.5 Graft and patient survival: long-term outcomes

Long-term outcomes for ABO-incompatible kidney transplants in terms of graft survival are generally promising, although some studies suggest that ABOi kidney grafts exhibit slightly lower survival rates compared to ABO-compatible (ABOc) grafts, particularly in the early post-transplant years. In our study, graft survival in ABOi patients was 100% after a mean follow-up of 4.6 ± 3 years. Similarly, Koo et al. (1) reported a graft survival rate of 92% in a cohort of 426 ABOi KTx patients after five years of follow-up, with no statistically significant difference observed between the low-titer ABOi group (≤1:64, n = 300) and the high-titer group (≥1:128, n = 126). These findings are further supported by Chung et al. (29), who found no significant difference in graft survival at one-year post-transplantation between ABOi KTx patients with high isoagglutinin titers (≥1:256) and those with low titers (≤1:128).

In our study, most ABOi grafts demonstrated stable renal function, with an average creatinine level of 121 ± 16 µmol/L after one year, and 138 ± 15 µmol/L after four years of follow-up. These results are consistent with those reported by Gan et al. (3), who observed an average creatinine level of 115 ± 37 µmol/L at one year and 143.8 ± 99 µmol/L after five years, alongside a graft survival rate of 90%. Similarly, a Spanish study by Oppenheimer et al. (14) found an average creatinine level of 115.8 ± 8.0 µmol/L at one year.

Despite these favorable outcomes, ABOi graft survival remains somewhat lower than that of ABOc grafts, particularly during the early post-transplant period. A meta-analysis by Scurt et al. (46), which included 65,063 transplant recipients, 7,098 of whom were ABOi patients, showed that three-year graft survival rates in ABOi kidney recipients were significantly lower compared to ABOc recipients. However, this difference diminishes after five years of follow-up, likely due to the elevated risk of acute rejection and infection in the early post-transplant phase. De Weerd et al. (13) also reported that ABOi graft survival was comparable to that of ABOc grafts from deceased donors, but slightly lower than that of ABOc grafts from living donors, especially in patients with isoagglutinin titers ≥ 1:128. The estimated glomerular filtration rate (GFR) at one year was, on average, 49.7 mL/min/1.73 m² in the ABOi group, compared to 55.1 mL/min/1.73 m² in the ABOc living donor group and 48.9 mL/min/1.73 m² in the ABOc deceased donor group. These results are consistent with those of Massie et al. (2), who demonstrated that ABOi kidney recipients experienced superior survival beyond 180 days post-transplant compared to matched candidates on the waiting list, although the mortality risk remained higher within the first 30 days post-transplantation.

In terms of patient survival, the outcomes of our study were equally favorable, with a long-term survival rate of 100% after a mean follow-up of 4.6 ± 3 years. In a previous study involving 44 ABOi and 44 ABOc KTx patients (6, 7), we also observed a 100% patient survival rate after a mean follow-up of 18 ± 14.8 months. These findings align with those of Gan et al. (3) and Koo et al. (1), who reported patient survival rates of 90% and 96%, respectively, in ABOi patients after five years of follow-up. In comparison, ABOc patients receiving a kidney graft from a deceased donor had a one-year survival rate of 97.3% and a five-year survival rate of 93%, while patients remaining on the waiting list exhibited survival rates of 97.6% and 90%, respectively (1). Thus, KTx ABOi patients benefit from superior survival compared to those on the transplant waiting list or those receiving ABOc grafts from deceased donors. These findings are corroborated by de Weerd et al. (13), who found that ABOi KTx patient survival was higher than that of ABOc recipients of deceased donor transplants [HR 0.69 (0.49-0.96)], and comparable to ABOc recipients of living donor transplants [HR 1.28 (0.90-1.81)]. The cumulative incidence of mortality with a functioning graft in ABOi patients was 3.0%, 6.4%, and 13.5% at 1, 5, and 10 years, compared to 1.6%, 7.0%, and 10.4% at 1, 5, and 10 years for ABOc transplant recipients.

## 5 Conclusion

The presence of elevated isoagglutinin titers should no longer be considered a barrier to ABO-incompatible (ABOi) kidney transplantation, thanks to advancements in desensitization protocols involving rituximab and apheresis. Our findings, alongside evidence from the literature, confirm that ABOi kidney transplants can achieve long-term patient survival rates comparable to, or even surpassing, those of ABO-compatible (ABOc) recipients of deceased donor grafts or patients remaining on the transplant waiting list. However, achieving these outcomes requires substantial expertise and resources, as effective management of ABOi transplants demands rigorous desensitization protocols, highly trained personnel in apheresis and immunology, and vigilant postoperative monitoring.

## Data Availability

The raw data supporting the conclusions of this article will be made available by the authors, without undue reservation.
